# Supplementary figures and images for: Does the skull Hounsfield unit predict shunt dependent hydrocephalus after decompressive craniectomy for traumatic acute subdural hematoma?
Source: PLoS One. 2020 Apr 30;15(4):e0232631. doi: 10.1371/journal.pone.0232631 (PMC7192490; doi:10.1371/journal.pone.0232631)

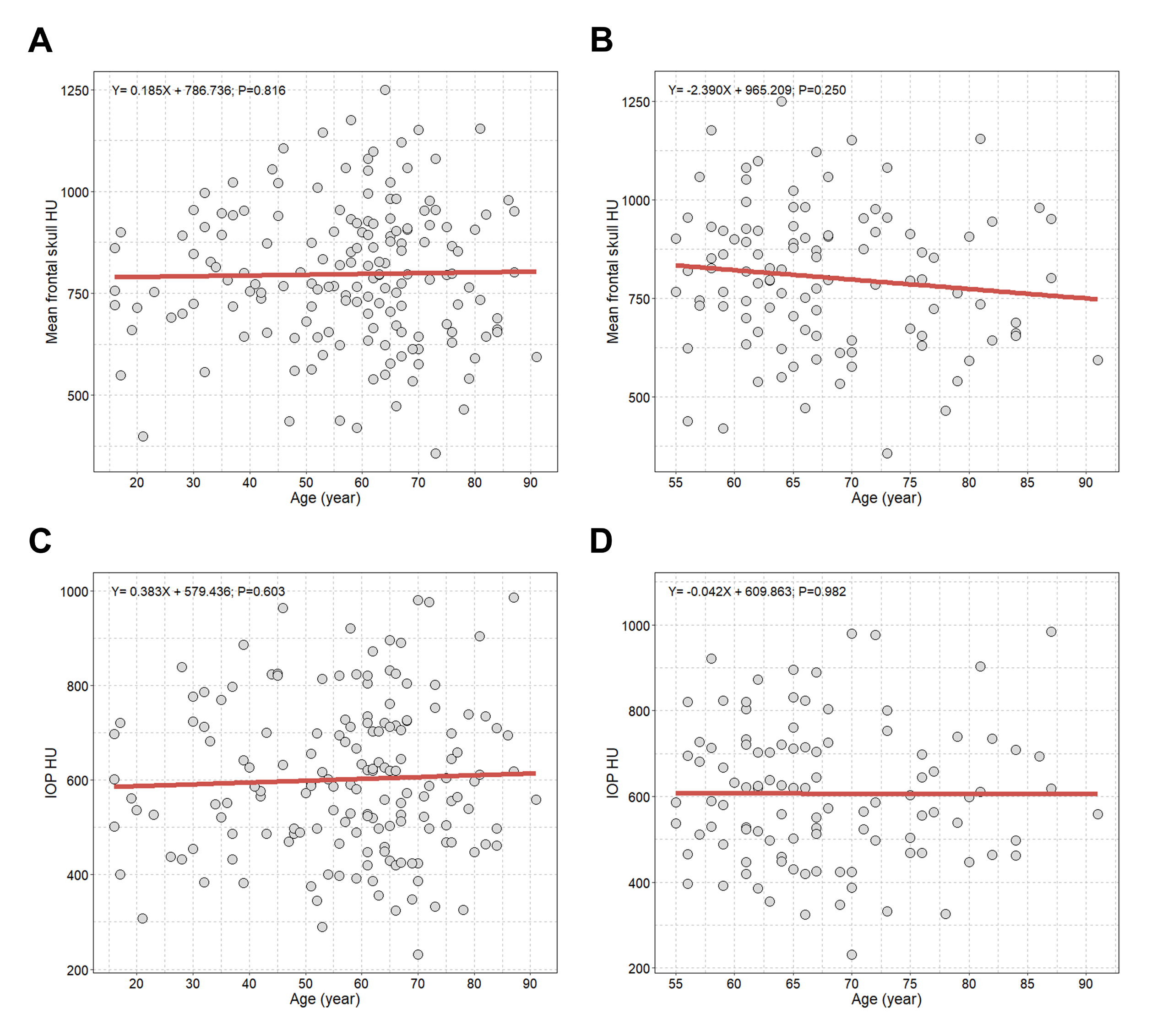

Supplement: S1 Fig — (A) association between age and mean frontal skull HU in all patients. (B) association between age and mean frontal skull HU in patients over 55 years of age. (C) association between age and IOP HU in all patients. (D) association between age and IOP HU in patients over 55 years of age. IOP = internal occipital protuberance; HU = Hounsfield unit. (TIF) [file pone.0232631.s001.tif]

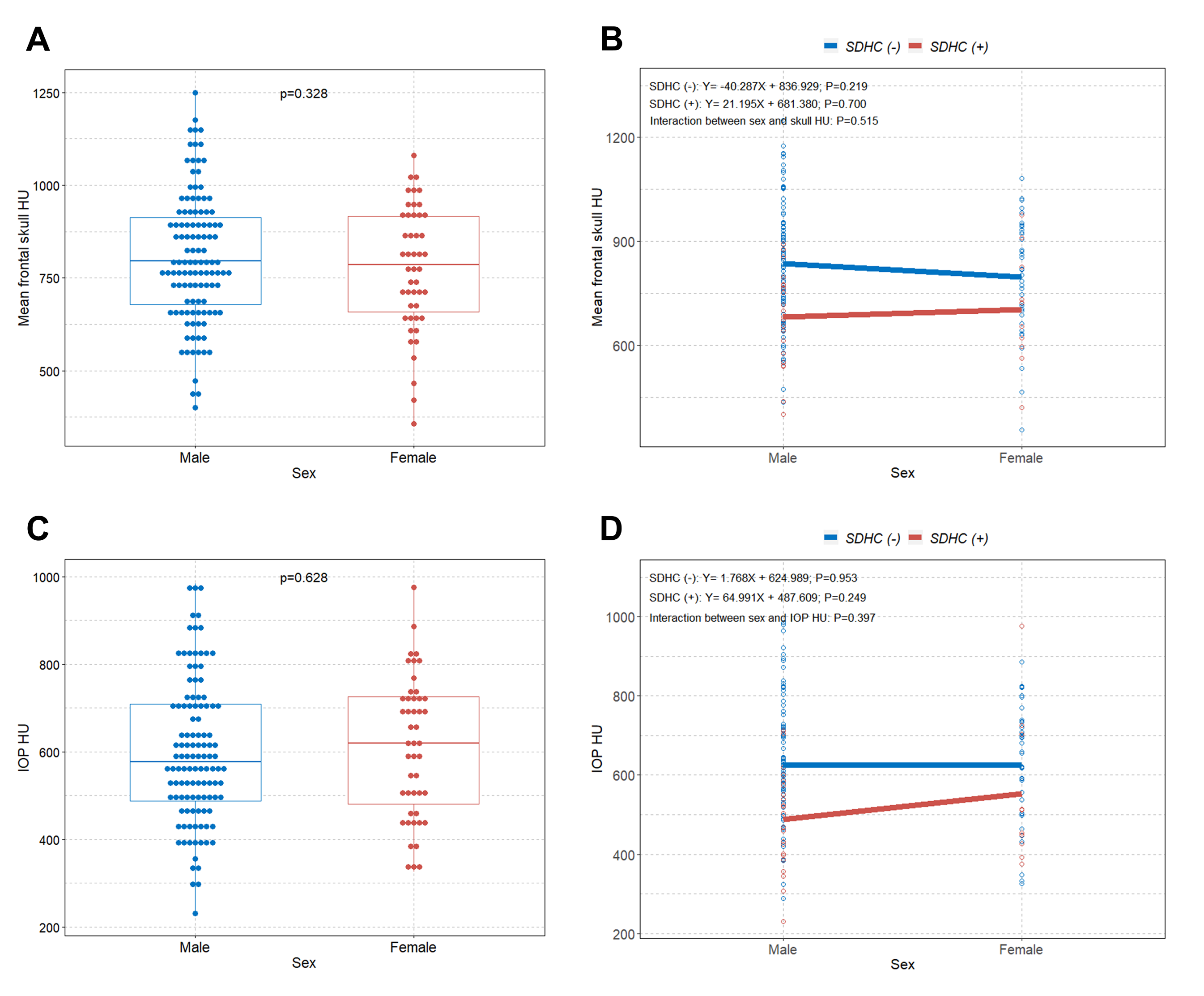

Supplement: S2 Fig — (A) boxplots with dot plots of mean frontal skull HU values according to the sex. (B) interaction between mean frontal skull HU and sex. (C) boxplots with dot plots of IOP HU values according to the sex. (D) interaction between IOP HU and sex. IOP = internal occipital protuberance; HU = Hounsfield unit; SDHC = shunt-dependent hydrocephalus. (TIF) [file pone.0232631.s002.tif]
