# Supplementary material for: Does the skull Hounsfield unit predict shunt dependent hydrocephalus after decompressive craniectomy for traumatic acute subdural hematoma?
Source: PLoS One. 2020 Apr 30;15(4):e0232631. doi: 10.1371/journal.pone.0232631 (PMC7192490; doi:10.1371/journal.pone.0232631)
Supplement: S1 Table — SDHC = shunt-dependent hydrocephalus; BMI = body mass index; HU = Hounsfield unit; IOP = internal occipital protuberance; GCS = Glasgow coma scale; SAH = subarachnoid hemorrhage; ICH = intracerebral hemorrhage; IVH = intraventricular hemorrhage; EDH = epidural hematoma; CI = confidence interval. (DOCX) [file pone.0232631.s003.docx]

|  | Multivariable logistic regression analysis | | |
| --- | --- | --- | --- |
| Variable | OR | 95%CI | P |
| Sex |  |  |  |
| Female (vs male) | 1.260 | 0.439 to 3.618 | 0.668 |
| Age (per 1-year increase) | 1.016 | 0.985 to 1.047 | 0.311 |
| BMI (per 1 BMI increase) | 1.043 | 0.927 to 1.175 | 0.482 |
| Mean frontal skull HU (per 1 HU increase) | 0.994 | 0.991 to 0.997 | <0.001 |
| Side of craniectomy |  |  |  |
| Left (vs right) | 0.467 | 0.165 to 1.326 | 0.153 |
| Bilateral (vs right) | 4.055 | 0.955 to 17.217 | 0.058 |
| Reoperation |  |  |  |
| Yes (vs no) | 1.620 | 0.348 to 7.538 | 0.538 |
| Midline shifting (mm) |  |  |  |
| > 10 and ≤ 20 (vs ≤ 10) | 1.636 | 0.461 to 5.810 | 0.447 |
| > 20 (vs ≤ 10) | 8.224 | 0.553 to 122.391 | 0.126 |
| Initial Glasgow coma scale  (per 1 score increase) | 0.843 | 0.639 to 1.113 | 0.228 |
| Traumatic SAH |  |  |  |
| Yes (vs no) | 0.475 | 0.141 to 1.603 | 0.230 |
| Traumatic ICH |  |  |  |
| Yes (vs no) | 1.400 | 0.446 to 4.394 | 0.564 |
| Traumatic IVH |  |  |  |
| Yes (vs no) | 0.560 | 0.144 to 2.169 | 0.401 |
| Traumatic EDH |  |  |  |
| Yes (vs no) | 2.204 | 0.295 to 16.478 | 0.441 |
| Skull fracture |  |  |  |
| Yes (vs no) | 0.546 | 0.104 to 2.871 | 0.475 |
| Hypertension | 0.576 | 0.175 to 1.896 | 0.364 |
| Diabetes | 0.261 | 0.047 to 1.431 | 0.122 |

Hosmer-Lemeshow goodness of fit test: χ^2^=12.968, P=0.113.

OR, odds ratio; CI, confidence interval; BMI, body mass index; HU, Hounsfield unit; SAH, subarachnoid hemorrhage; ICH, intracerebral hemorrhage; IVH, intraventricular hemorrhage; EDH, epidural hematoma.
