# Supplementary material for: Does the skull Hounsfield unit predict shunt dependent hydrocephalus after decompressive craniectomy for traumatic acute subdural hematoma?
Source: PLoS One. 2020 Apr 30;15(4):e0232631. doi: 10.1371/journal.pone.0232631 (PMC7192490; doi:10.1371/journal.pone.0232631)
Supplement: S2 Table — SDHC = shunt-dependent hydrocephalus; BMI = body mass index; HU = Hounsfield unit; IOP = internal occipital protuberance; GCS = Glasgow coma scale; SAH = subarachnoid hemorrhage; ICH = intracerebral hemorrhage; IVH = intraventricular hemorrhage; EDH = epidural hematoma; CI = confidence interval. (DOCX) [file pone.0232631.s004.docx]

|  | Multivariable logistic regression analysis | | |
| --- | --- | --- | --- |
| Variable | OR | 95%CI | P |
| Sex |  |  |  |
| Female (vs male) | 1.528 | 0.531 to 4.394 | 0.431 |
| Age (per 1-year increase) | 1.019 | 0.987 to 1.052 | 0.239 |
| BMI (per 1 BMI increase) | 1.038 | 0.923 to 1.167 | 0.533 |
| IOP HU (per 1 HU increase) | 0.993 | 0.989 to 0.997 | <0.001 |
| Side of craniectomy |  |  |  |
| Left (vs right) | 0.473 | 0.168 to 1.332 | 0.157 |
| Bilateral (vs right) | 4.934 | 1.139 to 21.373 | 0.033 |
| Reoperation |  |  |  |
| Yes (vs no) | 1.813 | 0.364 to 9.032 | 0.468 |
| Midline shifting (mm) |  |  |  |
| > 10 and ≤ 20 (vs ≤ 10) | 1.839 | 0.532 to 6.354 | 0.336 |
| > 20 (vs ≤ 10) | 6.280 | 0.400 to 98.641 | 0.191 |
| Initial Glasgow coma scale  (per 1 score increase) | 0.869 | 0.658 to 1.148 | 0.324 |
| Traumatic SAH |  |  |  |
| Yes (vs no) | 0.505 | 0.152 to 1.681 | 0.266 |
| Traumatic ICH |  |  |  |
| Yes (vs no) | 1.633 | 0.515 to 5.175 | 0.404 |
| Traumatic IVH |  |  |  |
| Yes (vs no) | 0.564 | 0.144 to 2.202 | 0.409 |
| Traumatic EDH |  |  |  |
| Yes (vs no) | 3.413 | 0.430 to 27.103 | 0.246 |
| Skull fracture |  |  |  |
| Yes (vs no) | 0.341 | 0.062 to 1.871 | 0.216 |
| Hypertension | 0.642 | 0.196 to 2.103 | 0.464 |
| Diabetes | 0.245 | 0.044 to 1.352 | 0.106 |

Hosmer-Lemeshow goodness of fit test: χ^2^=9.783, P=0.281.

OR, odds ratio; CI, confidence interval; BMI, body mass index; IOP, internal occipital protuberance; HU, Hounsfield unit; SAH, subarachnoid hemorrhage; ICH, intracerebral hemorrhage; IVH, intraventricular hemorrhage; EDH, epidural hematoma.
