# Supplementary material for: Does the skull Hounsfield unit predict shunt dependent hydrocephalus after decompressive craniectomy for traumatic acute subdural hematoma?
Source: PLoS One. 2020 Apr 30;15(4):e0232631. doi: 10.1371/journal.pone.0232631 (PMC7192490; doi:10.1371/journal.pone.0232631)
Supplement: Attachment — Submitted filename: Point-by-Point_Response_to_Reviewers.docx [file pone.0232631.s005.docx]

**Response to reviewers**

**Does the skull Hounsfield unit predict shunt dependent hydrocephalus after decompressive craniectomy for traumatic acute subdural hematoma?**

In-Suk Bae, Jae Min Kim, Jin Hwan Cheong, Je Il Ryu, Kyu-Sun Choi, Myung-Hoon Han

**Reviewer #2**

**The statistical methodology has two significant flaws. The first has to do with converting a numerical variable (HU) to a categorical one. The authors use ROC analysis to choose a threshold that optimally segregates hydro from non-hydro patients. They then use that threshold in the multivariable model. Since they’ve already proven this threshold significantly separates the two classes, it’s not surprising that the multivariable model finds the same thing. Why not leave HU as a numeric variable or ordinal variable, rather than bifurcating it? This was done with other numeric variables like age and BMI, so clearly the authors can do it. I am hopeful that this supplemental analysis (leaving HU numerical) won’t change the overall outcome of the study, but the authors should do it to reassure skeptical readers.**

Response: Thank you for your comment and we agree with you. We performed two separate multivariable logistic regressions with HU values as continuous covariates and presented the Supplemental Tables as you suggested as follows.

**S1 Table.** Multivariable logistic regression analysis for prediction of SDHC development according to potential predictive factors (adjusted for sex, age [continuous variable], BMI [continuous variable], mean frontal skull HU value [continuous variable], side of craniectomy, reoperation, midline shifting, initial GCS [continuous variable], traumatic SAH, ICH, IVH, EDH, skull fracture, hypertension, and diabetes). SDHC=shunt-dependent hydrocephalus; BMI=body mass index; HU=Hounsfield unit; IOP=internal occipital protuberance; GCS= Glasgow coma scale; SAH=subarachnoid hemorrhage; ICH=intracerebral hemorrhage; IVH=intraventricular hemorrhage; EDH=epidural hematoma; CI=confidence interval.

|  | Multivariable logistic regression analysis | | |
| --- | --- | --- | --- |
| Variable | OR | 95%CI | P |
| Sex |  |  |  |
| Female (vs male) | 1.260 | 0.439 to 3.618 | 0.668 |
| Age (per 1-year increase) | 1.016 | 0.985 to 1.047 | 0.311 |
| BMI (per 1 BMI increase) | 1.043 | 0.927 to 1.175 | 0.482 |
| Mean frontal skull HU (per 1 HU increase) | 0.994 | 0.991 to 0.997 | <0.001 |
| Side of craniectomy |  |  |  |
| Left (vs right) | 0.467 | 0.165 to 1.326 | 0.153 |
| Bilateral (vs right) | 4.055 | 0.955 to 17.217 | 0.058 |
| Reoperation |  |  |  |
| Yes (vs no) | 1.620 | 0.348 to 7.538 | 0.538 |
| Midline shifting (mm) |  |  |  |
| > 10 and ≤ 20 (vs ≤ 10) | 1.636 | 0.461 to 5.810 | 0.447 |
| > 20 (vs ≤ 10) | 8.224 | 0.553 to 122.391 | 0.126 |
| Initial Glasgow coma scale  (per 1 score increase) | 0.843 | 0.639 to 1.113 | 0.228 |
| Traumatic SAH |  |  |  |
| Yes (vs no) | 0.475 | 0.141 to 1.603 | 0.230 |
| Traumatic ICH |  |  |  |
| Yes (vs no) | 1.400 | 0.446 to 4.394 | 0.564 |
| Traumatic IVH |  |  |  |
| Yes (vs no) | 0.560 | 0.144 to 2.169 | 0.401 |
| Traumatic EDH |  |  |  |
| Yes (vs no) | 2.204 | 0.295 to 16.478 | 0.441 |
| Skull fracture |  |  |  |
| Yes (vs no) | 0.546 | 0.104 to 2.871 | 0.475 |
| Hypertension | 0.576 | 0.175 to 1.896 | 0.364 |
| Diabetes | 0.261 | 0.047 to 1.431 | 0.122 |

Hosmer-Lemeshow goodness of fit test: χ^2^=12.968, P=0.113.

OR, odds ratio; CI, confidence interval; BMI, body mass index; HU, Hounsfield unit; SAH, subarachnoid hemorrhage; ICH, intracerebral hemorrhage; IVH, intraventricular hemorrhage; EDH, epidural hematoma.

**S2 Table.** Multivariable logistic regression analysis for prediction of SDHC development according to potential predictive factors (adjusted for sex, age [continuous variable], BMI [continuous variable], IOP HU value [continuous variable], side of craniectomy, reoperation, midline shifting, initial GCS [continuous variable], traumatic SAH, ICH, IVH, EDH, skull fracture, hypertension, and diabetes). SDHC=shunt-dependent hydrocephalus; BMI=body mass index; HU=Hounsfield unit; IOP=internal occipital protuberance; GCS= Glasgow coma scale; SAH=subarachnoid hemorrhage; ICH=intracerebral hemorrhage; IVH=intraventricular hemorrhage; EDH=epidural hematoma; CI=confidence interval.

|  | Multivariable logistic regression analysis | | |
| --- | --- | --- | --- |
| Variable | OR | 95%CI | P |
| Sex |  |  |  |
| Female (vs male) | 1.528 | 0.531 to 4.394 | 0.431 |
| Age (per 1-year increase) | 1.019 | 0.987 to 1.052 | 0.239 |
| BMI (per 1 BMI increase) | 1.038 | 0.923 to 1.167 | 0.533 |
| IOP HU (per 1 HU increase) | 0.993 | 0.989 to 0.997 | <0.001 |
| Side of craniectomy |  |  |  |
| Left (vs right) | 0.473 | 0.168 to 1.332 | 0.157 |
| Bilateral (vs right) | 4.934 | 1.139 to 21.373 | 0.033 |
| Reoperation |  |  |  |
| Yes (vs no) | 1.813 | 0.364 to 9.032 | 0.468 |
| Midline shifting (mm) |  |  |  |
| > 10 and ≤ 20 (vs ≤ 10) | 1.839 | 0.532 to 6.354 | 0.336 |
| > 20 (vs ≤ 10) | 6.280 | 0.400 to 98.641 | 0.191 |
| Initial Glasgow coma scale  (per 1 score increase) | 0.869 | 0.658 to 1.148 | 0.324 |
| Traumatic SAH |  |  |  |
| Yes (vs no) | 0.505 | 0.152 to 1.681 | 0.266 |
| Traumatic ICH |  |  |  |
| Yes (vs no) | 1.633 | 0.515 to 5.175 | 0.404 |
| Traumatic IVH |  |  |  |
| Yes (vs no) | 0.564 | 0.144 to 2.202 | 0.409 |
| Traumatic EDH |  |  |  |
| Yes (vs no) | 3.413 | 0.430 to 27.103 | 0.246 |
| Skull fracture |  |  |  |
| Yes (vs no) | 0.341 | 0.062 to 1.871 | 0.216 |
| Hypertension | 0.642 | 0.196 to 2.103 | 0.464 |
| Diabetes | 0.245 | 0.044 to 1.352 | 0.106 |

Hosmer-Lemeshow goodness of fit test: χ^2^=9.783, P=0.281.

OR, odds ratio; CI, confidence interval; BMI, body mass index; IOP, internal occipital protuberance; HU, Hounsfield unit; SAH, subarachnoid hemorrhage; ICH, intracerebral hemorrhage; IVH, intraventricular hemorrhage; EDH, epidural hematoma.

We have added relevant sentences to the “Independent predictive factors for SDHC” section of the Results as follows.

**Results**

**Independent predictive factors for SDHC**

Furthermore, we performed two separate multivariable logistic regressions using the same covariates as above with HU values as continuous covariates (S1 and S2 Tables). Both mean frontal skull and IOP HU values that were entered as continuous covariates into the multivariable logistic model were significant independent predictors of SDHC in patients receiving cranioplasty after DC for traumatic acute SDH (OR, 0.994; P<0.001; OR, 0.994; P<0.001, respectively).

**The second problem is that there is no effort to test the fit of their multivariable model. It has a ton of variables and there’s no evidence they did anything to reduce the model. Having too many variables can easily mask significant interactions. An example of how to do this in R is given in this publication: https://www.ncbi.nlm.nih.gov/pmc/articles/PMC4828741/ . The authors need to convince us that there is no significant interaction among variables in the model they’ve created, and that we’re not missing an explanatory co-factor (like age correlating with HU, which it certainly does in all my patients).**

Response: Thank you for your comment and we agree with you. We performed the Hosmer-Lemeshow goodness of fit test on the logistic regression model as you suggested. Data seen below:

We have added the results of the Hosmer-Lemeshow goodness of fit test to Figure 4 as follows.

We also performed a linear regression analysis to assess the association between age and HU value. We present the results in S1 Fig. Unexpectedly, although a negative linear trend between age and HU values was observed in patients aged ≥55 years, there was no overall significant correlation between age and possible BMD in the study patients, as seen below.

A previous study from Korea reported that the prevalence of osteoporosis was 8.8% and 39.1% in men and women aged 50 years or higher (50 to 89 years), respectively (*Lee J, Lee S, Jang S, Ryu OH. Age-Related Changes in the Prevalence of Osteoporosis according to Gender and Skeletal Site: The Korea National Health and Nutrition Examination Survey 2008-2010. Endocrinol Metab. 2013;28: 180–191).* We think that the discrepancy of association between age and possible BMD in our study could be because our study included only trauma patients and relatively younger patients (mean age, 57 years) with women accounting for 29% of the study population. The interaction analysis showed that there was no significant effect of age on the HU value of SDHC development in the study patients.

We also added interaction plots and analyses that were performed using R software as follows.

**Figure 5**

**S2 Fig.**

We have also added relevant sentences to the Methods, Results, and Discussion sections as follows.

**Methods**

**Statistical methods**

Odds ratios (ORs) with 95% confidence intervals (CIs) were estimated using uni- and multivariate logistic regressions to determine the independent predictive factors for the development of SDHC in patients that received cranioplasty after DC for acute SDH. Sex, age (continuous variable), BMI (continuous variable), patients with simultaneous frontal skull HU ≤797.4 and IOP HU ≤586.5, side of craniectomy, reoperation, midline shifting, initial GCS (continuous variable), traumatic SAH, ICH, IVH, EDH, skull fracture, hypertension, and diabetes were entered into the multivariate model. The Hosmer-Lemeshow goodness of fit test was used to analyze the fit of the logistic regression model. Additional interaction analysis was performed using the R package, “effects”.

P-values less than 0.05 were considered statistically significant. All statistical analyses were performed using R software version 3.5.2 and SPSS for Windows, version 24.0 software (IBM, Chicago, IL).

**Results**

**Independent predictive factors for SDHC**

This multivariable logistic model was considered well-adjusted according to the Hosmer-Lemeshow test (P=0.806, χ^2^=4.538). Unexpectedly, although a negative linear trend between age and HU values was observed in patients aged ≥55 years, there was no overall significant correlation between age and possible BMD in the study patients (S1 Fig). However, because age is strongly related to BMD, we performed an additional interaction analysis of SDHC between skull and IOP HU values and age. However, we observed that there were no interactions of SDHC between the mean frontal skull HU, IOP HU, and simultaneous skull HU and IOP HU binary groups and age (P=0.507; P=0.284; P=0.721, respectively) (Fig. 5).

**Figure 5.** Interaction plots between skull and IOP HU values and age of SDHC. (A) between mean frontal skull HU and age. (B) between IOP HU and age. (C) between simultaneous skull HU and IOP HU binary groups and age. IOP=internal occipital protuberance; HU=Hounsfield unit; SDHC=shunt-dependent hydrocephalus.

In addition, we also tested the possible interaction between skull and IOP HU values and sex for SDHC development. Again, there were no interactions with respect to SDHC between the mean frontal skull HU and IOP HU and sex (P=0.515; P=0.397, respectively) (S2 Fig).

**Discussion**

Other possible predictive factors have been reported to be associated with posttraumatic hydrocephalus including intracranial pressure prior to decompression, subdural hygroma, low initial GCS score, increasing age, traumatic SAH, and traumatic IVH [31,31]. However, a previous study reported that interhemispheric hygromas after DC for severe head injury was the only independent prognostic factor for development of posttraumatic hydrocephalus [32].

Although there was a negative linear trend between age and HU value in patients aged ≥55 years, there was no overall correlation between age and HU value in the study patients. A previous study from Korea reported that the prevalence of osteoporosis was 8.8% and 39.1% in men and women aged 50 years or higher (50 to 89 years), respectively [33]. We think that the discrepancy of the association between age and possible BMD in our study could be because our study included only trauma patients and relatively younger patients (mean age, 57 years), with women only accounting for 29% of the study population. The interaction analysis showed that there was no significant effect of age on the HU value of SDHC development in the study patients.

Our study has several limitations.

**There is no mention of the data sharing for this paper, or that the data is publicly available, which is required for PLoS ONE submissions. This needs to be explicitly described and provided.**

Response: We added a data availability statement to the Methods section as follows.

**Methods**

**Data** **Availability Statement:** All relevant data are within the manuscript and its Supporting Information files.

**Minor issues:**

**The authors presumably mean “multivariable” regression and not “multivariate” regression. This is a common mistake. See** [**https://www.ncbi.nlm.nih.gov/pmc/articles/PMC3518362/**](https://www.ncbi.nlm.nih.gov/pmc/articles/PMC3518362/)

Response: Thank you for your comment and we apologize for our error. We have changed all expressions of “multivariate” with “multivariable” in the revised manuscript, as you suggested.

**Was there any missing data for any patient in the model? The paper should explicitly state that there was not, if there was not. If there WAS, they should absolutely document that.**

Response: Thank you for your comment. There were no missing data for any patient because all study patients were well followed up after decompressive craniectomy and underwent subsequent cranioplasty. We added a clarifying sentence to the Methods section as follows.

**Methods**

**Study design**

The remaining 162 patients were finally included in the study. All study patients were well followed up after DC and underwent subsequent cranioplasty; there were no missing data.

**More detail must be given about how patients were selected for shunts. The authors say “hydrocephalus symptoms”, which is far too vague.**

Response: Thank you for your comment and we agree. We have added relevant sentences to the Surgery and management section of the Methods as follows.

**Methods**

**Surgery and management**

V-P shunt was performed when the patients had hydrocephalus-related symptoms with the presence of ventricular enlargement on brain CT scans. Hydrocephalus-related symptoms for considering V-P shunt were defined as neurological deterioration and aggravated response to painful stimuli, especially in stuporous patients, and additional decline in the daily functions, gait disturbance, urinary incontinence, or confusion, especially in conscious patients. Simultaneous or staged V-P shunting was performed with or following cranioplasty.

**Reviewer #4**

**- it is interesting that there is significant correlation with skull HU and SDHC, but not age. One would expect that decreased skull HU would be very much age-dependent (as well as sex-dependent). This may be due to the fact that age odds ratios were measured per 1-year increase, while HU were measured in a binary fashion. The authors should discuss in the discussion why this expected relationship does not exist.**

Response: Thank you for your comment and we agree with you. We performed two separate multivariable logistic regressions with HU values as continuous covariates and presented the Supplemental Tables as you suggested as follows.

**S1 Table.** Multivariable logistic regression analysis for prediction of SDHC development according to potential predictive factors (adjusted for sex, age [continuous variable], BMI [continuous variable], mean frontal skull HU value [continuous variable], side of craniectomy, reoperation, midline shifting, initial GCS [continuous variable], traumatic SAH, ICH, IVH, EDH, skull fracture, hypertension, and diabetes). SDHC=shunt-dependent hydrocephalus; BMI=body mass index; HU=Hounsfield unit; IOP=internal occipital protuberance; GCS= Glasgow coma scale; SAH=subarachnoid hemorrhage; ICH=intracerebral hemorrhage; IVH=intraventricular hemorrhage; EDH=epidural hematoma; CI=confidence interval.

|  | Multivariable logistic regression analysis | | |
| --- | --- | --- | --- |
| Variable | OR | 95%CI | P |
| Sex |  |  |  |
| Female (vs male) | 1.260 | 0.439 to 3.618 | 0.668 |
| Age (per 1-year increase) | 1.016 | 0.985 to 1.047 | 0.311 |
| BMI (per 1 BMI increase) | 1.043 | 0.927 to 1.175 | 0.482 |
| Mean frontal skull HU (per 1 HU increase) | 0.994 | 0.991 to 0.997 | <0.001 |
| Side of craniectomy |  |  |  |
| Left (vs right) | 0.467 | 0.165 to 1.326 | 0.153 |
| Bilateral (vs right) | 4.055 | 0.955 to 17.217 | 0.058 |
| Reoperation |  |  |  |
| Yes (vs no) | 1.620 | 0.348 to 7.538 | 0.538 |
| Midline shifting (mm) |  |  |  |
| > 10 and ≤ 20 (vs ≤ 10) | 1.636 | 0.461 to 5.810 | 0.447 |
| > 20 (vs ≤ 10) | 8.224 | 0.553 to 122.391 | 0.126 |
| Initial Glasgow coma scale  (per 1 score increase) | 0.843 | 0.639 to 1.113 | 0.228 |
| Traumatic SAH |  |  |  |
| Yes (vs no) | 0.475 | 0.141 to 1.603 | 0.230 |
| Traumatic ICH |  |  |  |
| Yes (vs no) | 1.400 | 0.446 to 4.394 | 0.564 |
| Traumatic IVH |  |  |  |
| Yes (vs no) | 0.560 | 0.144 to 2.169 | 0.401 |
| Traumatic EDH |  |  |  |
| Yes (vs no) | 2.204 | 0.295 to 16.478 | 0.441 |
| Skull fracture |  |  |  |
| Yes (vs no) | 0.546 | 0.104 to 2.871 | 0.475 |
| Hypertension | 0.576 | 0.175 to 1.896 | 0.364 |
| Diabetes | 0.261 | 0.047 to 1.431 | 0.122 |

Hosmer-Lemeshow goodness of fit test: χ^2^=12.968, P=0.113.

OR, odds ratio; CI, confidence interval; BMI, body mass index; HU, Hounsfield unit; SAH, subarachnoid hemorrhage; ICH, intracerebral hemorrhage; IVH, intraventricular hemorrhage; EDH, epidural hematoma.

**S2 Table.** Multivariable logistic regression analysis for prediction of SDHC development according to potential predictive factors (adjusted for sex, age [continuous variable], BMI [continuous variable], IOP HU value [continuous variable], side of craniectomy, reoperation, midline shifting, initial GCS [continuous variable], traumatic SAH, ICH, IVH, EDH, skull fracture, hypertension, and diabetes). SDHC=shunt-dependent hydrocephalus; BMI=body mass index; HU=Hounsfield unit; IOP=internal occipital protuberance; GCS= Glasgow coma scale; SAH=subarachnoid hemorrhage; ICH=intracerebral hemorrhage; IVH=intraventricular hemorrhage; EDH=epidural hematoma; CI=confidence interval.

|  | Multivariable logistic regression analysis | | |
| --- | --- | --- | --- |
| Variable | OR | 95%CI | P |
| Sex |  |  |  |
| Female (vs male) | 1.528 | 0.531 to 4.394 | 0.431 |
| Age (per 1-year increase) | 1.019 | 0.987 to 1.052 | 0.239 |
| BMI (per 1 BMI increase) | 1.038 | 0.923 to 1.167 | 0.533 |
| IOP HU (per 1 HU increase) | 0.993 | 0.989 to 0.997 | <0.001 |
| Side of craniectomy |  |  |  |
| Left (vs right) | 0.473 | 0.168 to 1.332 | 0.157 |
| Bilateral (vs right) | 4.934 | 1.139 to 21.373 | 0.033 |
| Reoperation |  |  |  |
| Yes (vs no) | 1.813 | 0.364 to 9.032 | 0.468 |
| Midline shifting (mm) |  |  |  |
| > 10 and ≤ 20 (vs ≤ 10) | 1.839 | 0.532 to 6.354 | 0.336 |
| > 20 (vs ≤ 10) | 6.280 | 0.400 to 98.641 | 0.191 |
| Initial Glasgow coma scale  (per 1 score increase) | 0.869 | 0.658 to 1.148 | 0.324 |
| Traumatic SAH |  |  |  |
| Yes (vs no) | 0.505 | 0.152 to 1.681 | 0.266 |
| Traumatic ICH |  |  |  |
| Yes (vs no) | 1.633 | 0.515 to 5.175 | 0.404 |
| Traumatic IVH |  |  |  |
| Yes (vs no) | 0.564 | 0.144 to 2.202 | 0.409 |
| Traumatic EDH |  |  |  |
| Yes (vs no) | 3.413 | 0.430 to 27.103 | 0.246 |
| Skull fracture |  |  |  |
| Yes (vs no) | 0.341 | 0.062 to 1.871 | 0.216 |
| Hypertension | 0.642 | 0.196 to 2.103 | 0.464 |
| Diabetes | 0.245 | 0.044 to 1.352 | 0.106 |

Hosmer-Lemeshow goodness of fit test: χ^2^=9.783, P=0.281.

OR, odds ratio; CI, confidence interval; BMI, body mass index; IOP, internal occipital protuberance; HU, Hounsfield unit; SAH, subarachnoid hemorrhage; ICH, intracerebral hemorrhage; IVH, intraventricular hemorrhage; EDH, epidural hematoma.

We have added relevant sentences to the “Independent predictive factors for SDHC” section of the Results as follows.

**Results**

**Independent predictive factors for SDHC**

Furthermore, we performed two separate multivariable logistic regressions using the same covariates as above with HU values as continuous covariates (S1 and S2 Tables). Both mean frontal skull and IOP HU values that were entered as continuous covariates into the multivariable logistic model were significant independent predictors of SDHC in patients receiving cranioplasty after DC for traumatic acute SDH (OR, 0.994; P<0.001; OR, 0.994; P<0.001, respectively).

We also performed a linear regression analysis to assess the association between age and HU value. We present the results in S1 Fig. Unexpectedly, although a negative linear trend between age and HU values was observed in patients aged ≥55 years, there was no overall significant correlation between age and possible BMD in the study patients, as seen below.

A previous study from Korea reported that the prevalence of osteoporosis was 8.8% and 39.1% in men and women aged 50 years or higher (50 to 89 years), respectively (*Lee J, Lee S, Jang S, Ryu OH. Age-Related Changes in the Prevalence of Osteoporosis according to Gender and Skeletal Site: The Korea National Health and Nutrition Examination Survey 2008-2010. Endocrinol Metab. 2013;28: 180–191).* We think that the discrepancy of association between age and possible BMD in our study could be because our study included only trauma patients and relatively younger patients (mean age, 57 years) with women accounting for 29% of the study population. The interaction analysis showed that there was no significant effect of age on the HU value of SDHC development in the study patients.

We also added interaction plots and analyses that were performed using R software as follows.

**Figure 5**

**S2 Fig.**

We have also added relevant sentences to the Methods, Results, and Discussion sections as follows.

**Methods**

**Statistical methods**

Odds ratios (ORs) with 95% confidence intervals (CIs) were estimated using uni- and multivariate logistic regressions to determine the independent predictive factors for the development of SDHC in patients that received cranioplasty after DC for acute SDH. Sex, age (continuous variable), BMI (continuous variable), patients with simultaneous frontal skull HU ≤797.4 and IOP HU ≤586.5, side of craniectomy, reoperation, midline shifting, initial GCS (continuous variable), traumatic SAH, ICH, IVH, EDH, skull fracture, hypertension, and diabetes were entered into the multivariate model. The Hosmer-Lemeshow goodness of fit test was used to analyze the fit of the logistic regression model. Additional interaction analysis was performed using the R package, “effects”.

P-values <0.05 were considered statistically significant. All statistical analyses were performed using R software version 3.5.2 and SPSS for Windows, version 24.0 software (IBM, Chicago, IL).

**Results**

**Independent predictive factors for SDHC**

Unexpectedly, although a negative linear trend between age and HU values was observed in patients aged ≥55 years, there was no overall significant correlation between age and possible BMD in the study patients (S1 Fig). However, because age is strongly related to BMD, we performed an additional interaction analysis of SDHC between skull and IOP HU values and age. However, we observed that there were no interactions of SDHC between the mean frontal skull HU, IOP HU, and simultaneous skull HU and IOP HU binary groups and age (P=0.507; P=0.284; P=0.721, respectively) (Fig. 5).

**Figure 5.** Interaction plots between skull and IOP HU values and age of SDHC. (A) between mean frontal skull HU and age. (B) between IOP HU and age. (C) between simultaneous skull HU and IOP HU binary groups and age. IOP=internal occipital protuberance; HU=Hounsfield unit; SDHC=shunt-dependent hydrocephalus.

In addition, we also tested the possible interaction between skull and IOP HU values and sex for SDHC development. Again, there were no interactions with respect to SDHC between the mean frontal skull HU and IOP HU and sex (P=0.515; P=0.397, respectively) (S2 Fig).

**Discussion**

Other possible predictive factors have been reported to be associated with posttraumatic hydrocephalus including intracranial pressure prior to decompression, subdural hygroma, low initial GCS score, increasing age, traumatic SAH, and traumatic IVH [31,31]. However, a previous study reported that interhemispheric hygromas after DC for severe head injury was the only independent prognostic factor for development of posttraumatic hydrocephalus [32].

Although there was a negative linear trend between age and HU value in patients aged ≥55 years, there was no overall correlation between age and HU value in the study patients. A previous study from Korea reported that the prevalence of osteoporosis was 8.8% and 39.1% in men and women aged 50 years or higher (50 to 89 years), respectively [33]. We think that the discrepancy of the association between age and possible BMD in our study could be because our study included only trauma patients and relatively younger patients (mean age, 57 years), with women only accounting for 29% of the study population. The interaction analysis showed that there was no significant effect of age on the HU value of SDHC development in the study patients.

Our study has several limitations.

**- "shunt dependent" is difficult to assess, especially in post-traumatic hydrocephalus as sometimes the indications for shunt placement vary by practitioner. The authors should add additional information on how they determined the need for shunt placement, and what they consider to be hydrocephalus-related symptoms (ie, depressed level of consciousness only, or subtler symptoms like headache)**

Response: Thank you for your comment and we agree. We have added relevant sentences to the Surgery and management section of the Methods as follows.

**Methods**

**Surgery and management**

V-P shunt was performed when the patients had hydrocephalus-related symptoms with the presence of ventricular enlargement on brain CT scans. Hydrocephalus-related symptoms for considering V-P shunt were defined as neurological deterioration and aggravated response to painful stimuli, especially in stuporous patients, and additional decline in the daily functions, gait disturbance, urinary incontinence, or confusion, especially in conscious patients. Simultaneous or staged V-P shunting was performed with or following cranioplasty.
